# Dietary teasaponin ameliorates alteration of gut microbiota and cognitive decline in diet-induced obese mice

**DOI:** 10.1038/s41598-017-12156-2

**Published:** 2017-09-22

**Authors:** Sen Wang, Xu-Feng Huang, Peng Zhang, Kelly A. Newell, Hongqin Wang, Kuiyang Zheng, Yinghua Yu

**Affiliations:** 1 Illawarra Health and Medical Research Institute, School of Medicine, University of Wollongong, Northfield Avenue, Wollongong, NSW 2522 Australia; 2 Department of Endocrinology and Metabolism, The people’s hospital of Suzhou National New & Hi-Tech Industrial development Zone, Suzhou, Jiangsu 215011 China; 3 Department of Pathogen Biology and Immunology, Jiangshu Key Laboratory of Immunity and Metabolism, Xuzhou Medical University, Xuzhou, Jiangsu 221004 China

## Abstract

A high-fat (HF) diet alters gut microbiota and promotes obesity related inflammation and cognitive impairment. Teasaponin is the major active component of tea, and has been associated with anti-inflammatory effects and improved microbiota composition. However, the potential protective effects of teasaponin, against HF diet-induced obesity and its associated alteration of gut microbiota, inflammation and cognitive decline have not been studied. In this study, obesity was induced in C57BL/6 J male mice by feeding a HF diet for 8 weeks, followed by treatment with oral teasaponin (0.5%) mixed in HF diet for a further 6 weeks. Teasaponin treatment prevented the HF diet-induced recognition memory impairment and improved neuroinflammation, gliosis and brain-derived neurotrophic factor (BDNF) deficits in the hippocampus. Furthermore, teasaponin attenuated the HF diet-induced endotoxemia, pro-inflammatory macrophage accumulation in the colon and gut microbiota alterations. Teasaponin also improved glucose tolerance and reduced body weight gain in HF diet-induced obese mice. The behavioral and neurochemical improvements suggest that teasaponin could limit unfavorable gut microbiota alterations and cognitive decline in HF diet-induced obesity.

## Introduction

Obesity increases the incidence of insulin resistance, type 2 diabetes, and cognitive decline in neurodegenerative diseases such as vascular dementia and Alzheimer’s disease (AD)^1,2^. Obesity is characterized by low grade systemic inflammation, which is associated with an alteration of gut microbiota and brain function^3^. Empirical evidence has linked a high-fat (HF) diet with alterations of gut microbiota^4^ and impairments in cognition, including a decline in recognition memory in both animal and human studies^5,6^. The hippocampus plays an important role in improving recognition memory^7–9^. Lesions in the hippocampus severely disrupt object recognition^7^, object-in-place memory^10^, and temporal order recognition memory^11^ in rodent studies.

Previous studies have provided compelling evidence to suggest an association between the gut microbiota, HF diet and body weight regulation^4,12^. Firmicutes and Bacteriodetes account for more than 90% of the total gut microbiota^13^. An 8-week HF diet increased Firmicutes and reduced Bacteriodetes in mice^4^. Consistent with animal models, a similar difference of an increased ratio of Firmicutes/Bacteroidetes in the gut microbiota has also been reported in obese humans^14^. Moreover, increased fat intake has been strongly correlated with increased plasma lipopolysaccharide (LPS), the endotoxin which is a major component of the outer membrane in Gram-negative bacteria^10^. In HF diet-induced obesity, endotoxemia has been linked with alterations of gut microbiota and increased intestinal permeability^11^. Currently, obesity and the related cognitive decline are associated with microbiota alterations and low-grade systemic and central inflammation^9,10^, which contribute to interruption of the gut-brain axis^3^.

It is known that endotoxemia can trigger systemic inflammation including neuroinflammation, and cognitive impairment^15^. For example, systemic LPS administration activated microglia and increased expression of pro-inflammatory factors in the hippocampus of mice^16^. Microglia and astrocytes are the principal immune cells in the central nervous system (CNS). The activation of microglia promotes the release of pro-inflammatory cytokines, which contribute to the neurodegenerative process^15^. Microglia and astrocytes express numerous members of the Toll-like receptor (TLR) family^17^, which recognize conserved microbial motifs expressed by a wide array of pathogens. For example, LPS binds to TLR4 mediated by myeloid differentiation primary-response protein 88 (MyD88), and activates c-Jun N-terminal kinase (JNK) and nuclear factor-kappa B (NFκB), two important inflammatory signaling molecules^17^. The activation of the TLR4-MyD88-JNK/NFκB signaling pathway leads to the production of pro-inflammatory cytokines, such as interleukin-1β (IL-1β), interleukin-6 (IL-6), and tumor necrosis factor-α (TNF-α), and contributes to the development of neurodegenerative diseases^18–21^. Adult hippocampal neurogenesis and its related learning and memory are influenced negatively by microglial activation and neuroinflammation^18^. Furthermore, intestinal microbiota affect central levels of brain-derived neurotropic factor (BDNF) and cognitive behavior. Administration of oral antimicrobials to SPF mice transiently alter the composition of intestinal microbiota and increase hippocampal expression of BDNF^19^.

Tea has been cultivated for thousands of years and used for beverage and medicinal purposes. Previous studies have found tea can prevent obesity and abnormal glucose and lipid metabolism^20^. Recently, research on tea has shown it possesses anti-inflammatory properties, and improves microbiota and cognition^21–25^. Phenolics and saponin are the two major active components of tea extract. Most research to date has focused on the interaction of dietary polyphenols and intestinal microbiota^26–28^. However, the effects of teasaponin on the modulation of the intestinal microbiota and cognition remain poorly investigated and understood. This study used a chronic HF diet-induced obese mouse model to assess the ability of dietary teasponin to regulate the gut-brain axis and ultimately prevent cognitive impairment, glucose intolerance and body weight gain. The gut microbiota, endotoxinemia, colonic macrophage shift, hippocampal inflammation and gliosis, recognition memory, body weight gain, and glucose tolerance were examined in these mice.

## Results

### Teasaponin reversed the alteration of gut microbiota and systemic inflammation induced by a HF diet in obese mice

**Figure 1:**
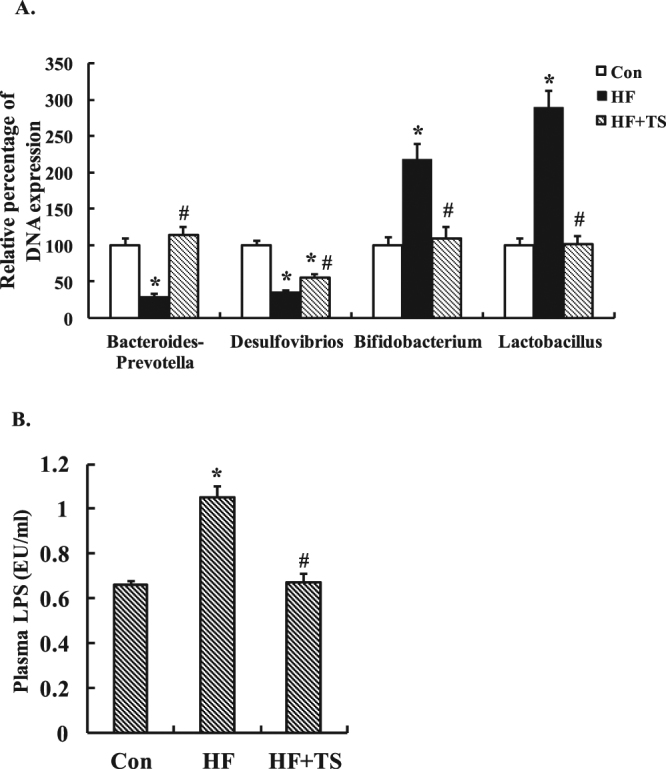
(**A**) Bacteroides-Prevotella, Desulfovibrios, Bifidobacterium and Lactobacillus DNA expressions in gut microbiota of the control group (Con), high-fat diet group (HF), and HF with teasaponin treatment group (HF + TS) (n = 12 per group). *p < 0.05 compared to the Con group, ^#^ *p* < 0.05 compared to the HF group, values are means ± SEM. (**B**) The plasma LPS level of control group (Con), high-fat diet group (HF), and HF with teasaponin treatment group (HF + TS) (n = 12 per group).

To investigate the effect of high-fat diet on intestinal microbiota and whether teasaponin could reverse this effect, qRT-PCR was used to evaluate the abundance of several vital species of gut flora in the cecal content, including *Bacteroides-Prevotella* spp., *Desulfovibrios* spp., *Bifidobacterium* spp. and *Lactobacillus* spp. The HF diet significantly decreased the amount of *Bacteroides-Prevotella* spp. (*p* = 0.016) and *Desulfovibrios* spp. DNA (*p* < 0.001), and increased *Bifidobacterium* spp. (*p* = 0.002) and *Lactobacillus* spp. DNA (*p* = 0.001) compared with the control mice (Fig. 1A). A 6-week teasaponin oral treatment prevented the alteration of gut microbiota induced by the HF diet. The amount of *Bacteroides-Prevotella* spp. DNA and *Desulfovibrios* spp. DNA were significantly increased in the HF + TS group compared to the HF group (*p* = 0.007, *p* = 0.013 respectively). The amount of *Bacteroides-Prevotella* spp. DNA in the HF + TS group was similar to the control mice (*p* = 0.488), while the amount of *Desulfovibrios* spp. DNA in the HF + TS group was still significantly lower than the control mice (*p* < 0.001). Meanwhile, the teasaponin treatment also prevented the HF diet-induced alteration of the amount of *Bifidobacterium* spp. DNA (*p* = 0.003) and *Lactobacillus* spp. DNA (*p* = 0.001) in the HF + TS group compared to the HF group. There was no statistical difference between the HF + TS group and the control group in *Lactobacillus* spp. DNA (*p* = 0.992) and *Bifidobacterium* spp. DNA (*p* = 0.701) (Fig. 1A).

To further determine whether teasaponin also decreased systemic inflammation with improving gut microbiota, the plasma concentration of LPS was measured. Teasaponin prevented an increase of plasma LPS induced by the HF diet after the 6-week treatment (Control: 0.66 ± 0.03 EU/ml, HF: 1.05 ± 0.08 EU/ml, HF + TS: 0.67 ± 0.04 EU/ml, *p* = 0.001). There was no difference between the control and the HF + TS groups in the plasma LPS concentrations (*p* = 0.805) (Fig. 1B).

### Teasaponin reduced M1 macrophage accumulation in the colon of HF diet-induced obese mice

**Figure 2:**
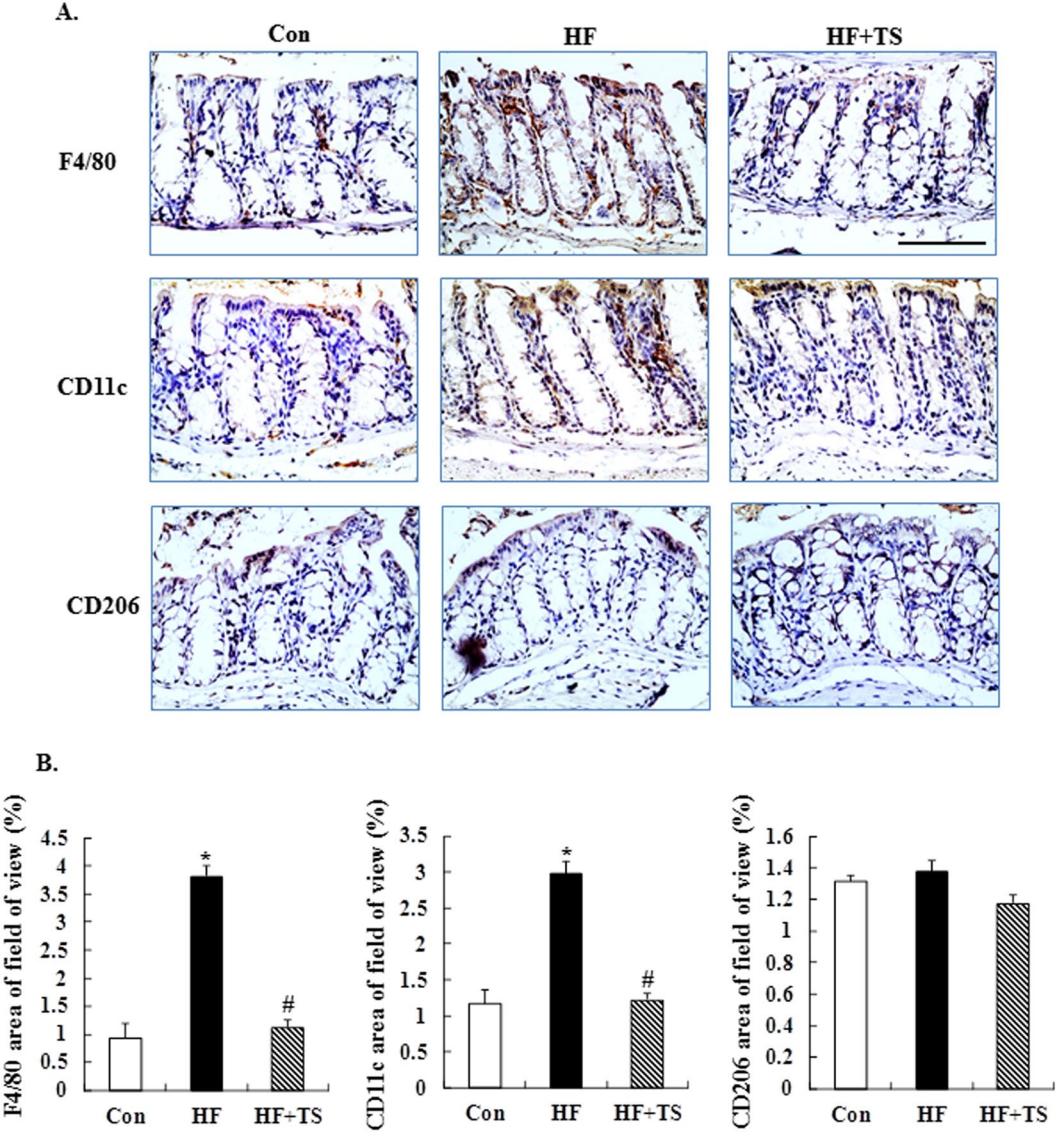
The expression of F4/80, CD11c, and CD206 macrophages in the colon. (**A**) Immunohistochemical staining, scale bar = 100 μm. (**B**) Quantification of the F4/80, CD11c, and CD206-positive areas (%). *p < 0.05 compared to the Con group, ^#^ *p* < 0.05 compared to the HF group, values are means ± SEM.

To investigate the effect of teasaponin on macrophage accumulation in the colon of HF diet-induced obese mice, macrophages were stained with F4/80 antibody. The positive immunoreactivity of F4/80 was significantly increased in the colon of obese mice (*p* < 0.001), while it was ameliorated by the teasaponin treatment (*p* < 0.001) (Fig. 2A and B). Furthermore, the types of macrophages were characterized. CD11c was used to detect M1 macrophages, which produce pro-inflammatory cytokines, and CD206 was used to detect M2 macrophages which produce anti-inflammatory cytokines. Teasaponin significantly reduced the CD11c positive staining (*p* < 0.001), but there was no significant difference in the CD206 staining of the colon among the control group, the HF group and the HF + TS group (Fig. 2A and B).

### Teasaponin suppressed the hippocampal inflammation in HF diet-induced obese mice

**Figure 3:**
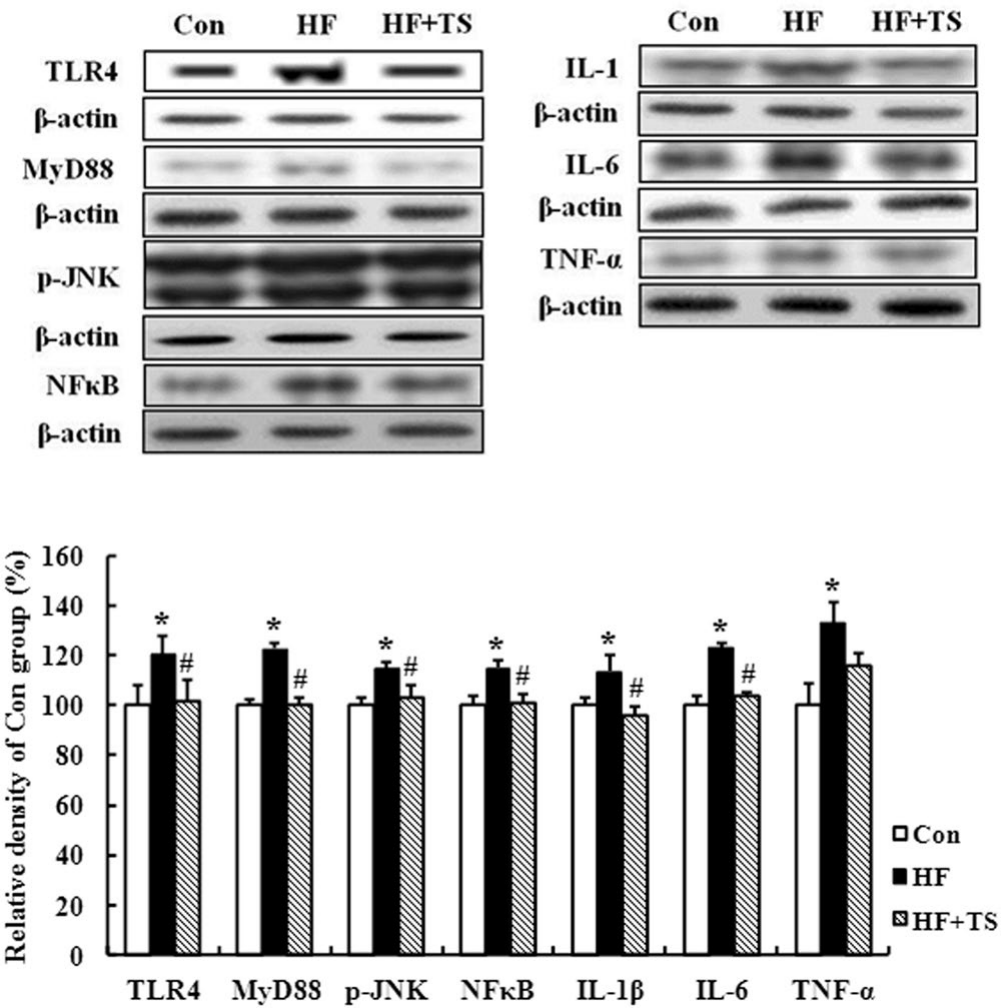
Protein expression levels of TLR4, MyD88, p-JNK, NFκB, IL-1β, IL-6, and TNF-α in the hippocampus of the control group (Con), high-fat diet group (HF), and HF with teasaponin treatment group (HF + TS) (n = 12 per group). *p < 0.05 compared to the Con group, ^#^ *p* < 0.05 compared to the HF group, values are means ± SEM.

To investigate whether LPS (endotoxemia) could be related to hippocampal inflammation, the TLR4-My88-NFκB/JNK signaling pathway and pro-inflammatory cytokines (TNF-α, IL-6 and IL-1β) were examined in hippocampal tissue. Western blotting revealed that the TLR4 (*p* = 0.036) and MyD88 (*p* < 0.001) levels in the hippocampus were significantly higher in the HF group than the control group. The TLR4 and MyD88 levels were significantly lower in the HF + TS group compared to the HF group (*p* = 0.032, *p* < 0.001 respectively), with the teasaponin treatment restoring the TLR4 and MyD88 levels to control levels (Fig. 3). Furthermore, the p-JNK (*p* < 0.001) and NFκB (*p* = 0.005) levels were significantly higher in the HF group compared to the control group, while the teasaponin treatment prevented an increase of p-JNK and NFκB (*p* < 0.001, *p* = 0.008) (Fig. 3). Levels of pro-inflammatory cytokines, IL-1β (*p* = 0.015), IL-6 (*p* = 0.006) and TNF-α (*p* = 0.009) were significantly higher in the HF group compared to the control group (Fig. 3). Teasaponin significantly decreased IL-1β (*p* = 0.003) and IL-6 (*p* = 0.015) levels compared to the HF group, with the HF + TS group showing no difference in IL-1β and IL-6 levels compared the control group.

### Teasaponin ameliorated microglial and astrocyte accumulation in the hippocampus of HF diet-induced obese mice

**Figure 4:**
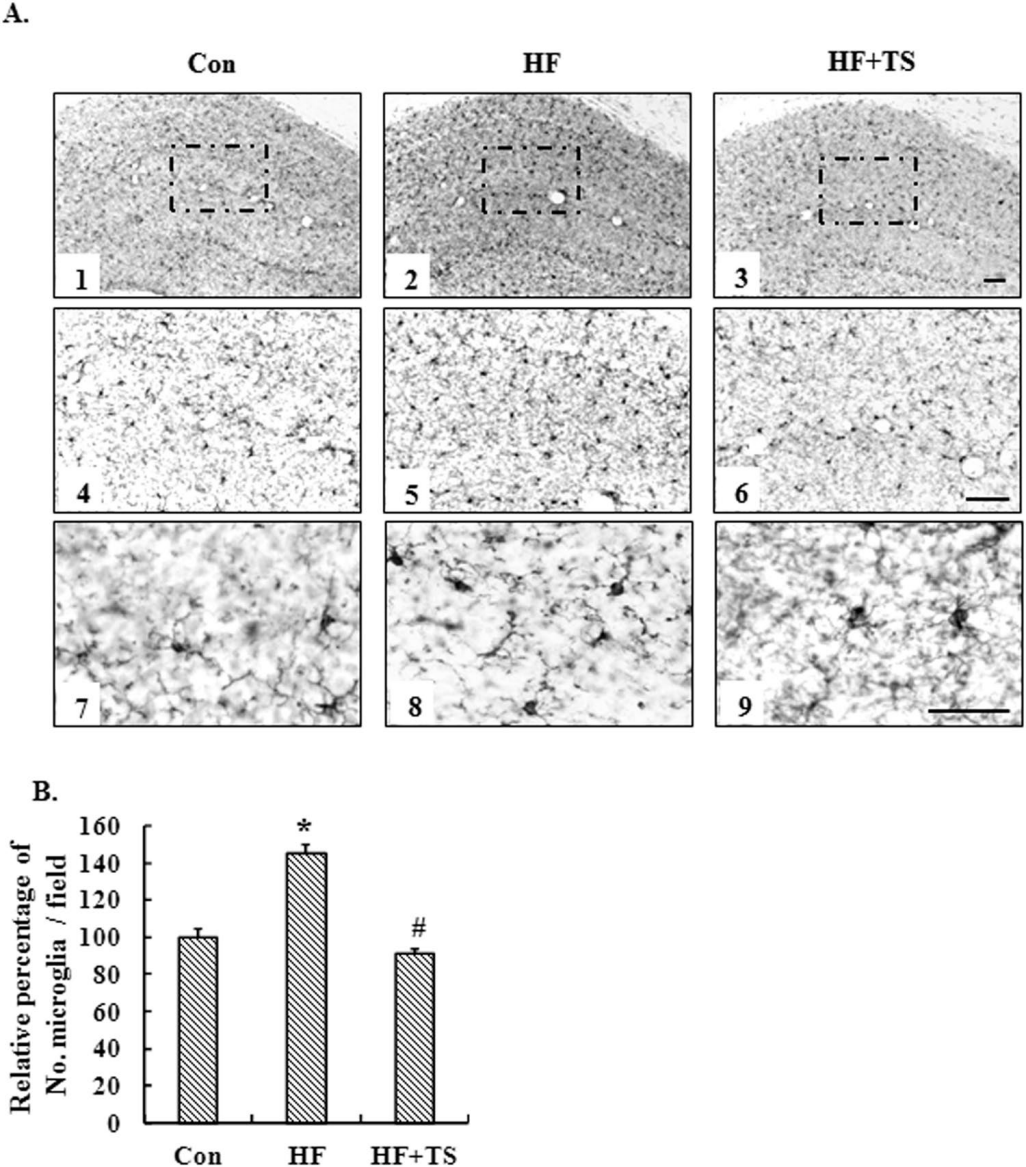
(**A**) Microglia accumulation in the hippocampus of the control group (Con), high-fat diet group (HF) and HF with teasaponin treatment group (HF + TS) shown at ×5 (A1-3), ×10 (A4-6) and ×40 (A7-9) magnification; Scale bar = 20 μm. (**B**) Relative percentage of No. microglia in the hippocampus (%). *p < 0.05 compared to the Con group, ^#^ *p* < 0.05 compared to the HF group, values are means ± SEM.

**Figure 5:**
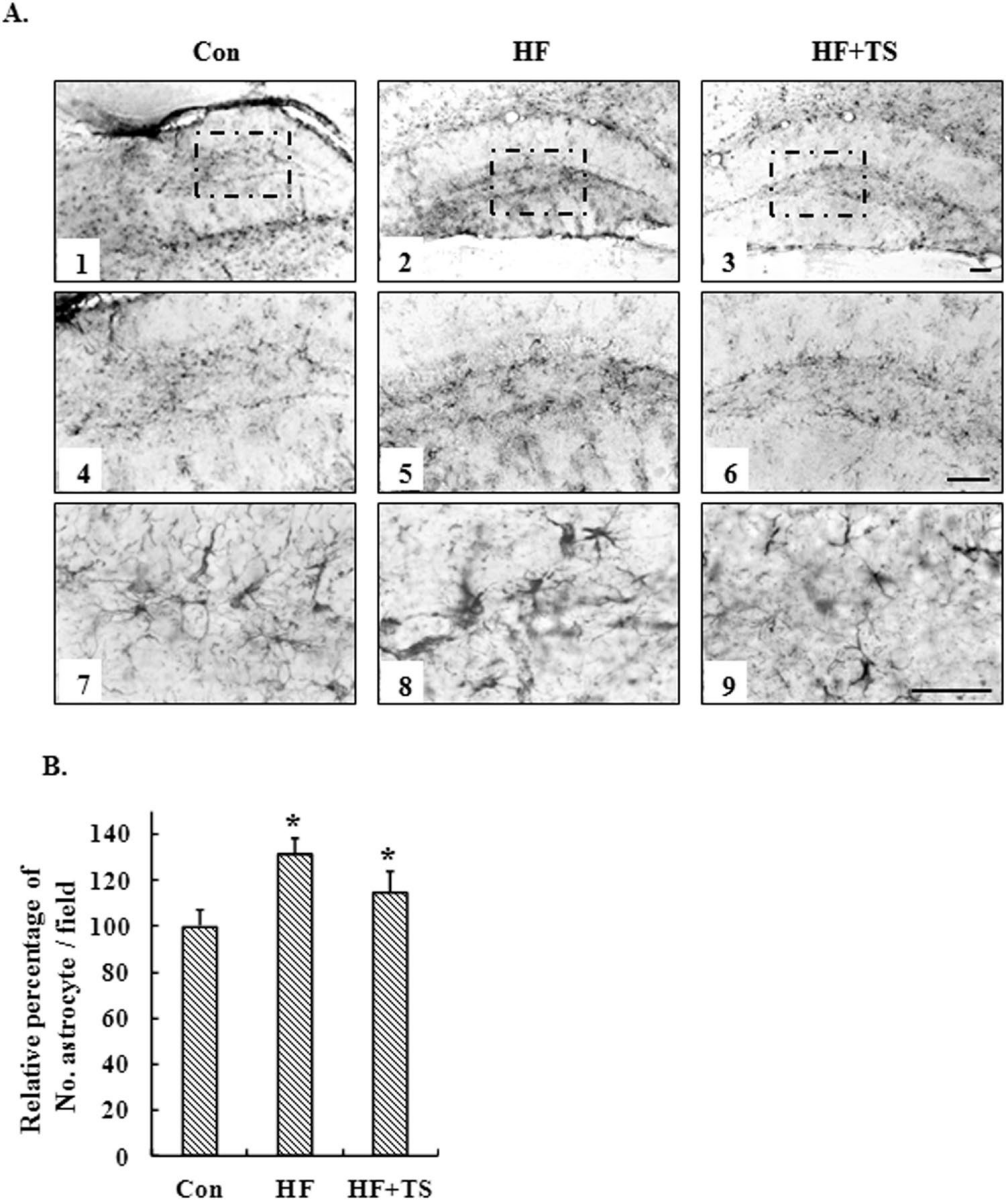
(**A**) Astrocyte accumulation in the hippocampus of the control group (Con), high-fat diet group (HF) and HF with teasaponin treatment group (HF + TS) shown at ×5 (A1-3), ×10 (A4-6) and ×40 (A7-9) magnification; Scale bar = 20 μm. (**B**) Relative percentage of No. astrocyte in the hippocampus (%). *p < 0.05 compared to the Con group, ^#^ *p* < 0.05 compared to the HF group, values are means ± SEM.

Activation, recruitment, and proliferation of microglia and astrocytes are hallmarks of the brain response to neuronal injury. We therefore assessed the expression of a microglial cell-specific marker, Iba-1 and an astrocyte activation marker, GFAP, in the hippocampus to investigate the effects of a HF diet on inflammatory responses and to examine whether teasaponin mitigates the activation and infiltration of glial cells in the brain. Immunohistochemistry revealed the number of hippocampal microglia in the HF group increased nearly 50% compared to the control group (*p* < 0.001), while teasaponin significantly ameliorated microglial accumulation in the hippocampus of high-fat diet-induced obese mice after 6 weeks treatment (*p* < 0.001) (Fig. 4). Microglial number in the HF + TS group was similar to the control group (*p* = 0.214). Meanwhile, the HF diet increased the number of astrocytes in the hippocampus of obese mice by nearly 25% compared to the control mice fed lab chow (*p* = 0.002). Teasaponin, to some degree, ameliorated astrocyte accumulation in the hippocampus of the HF group, but there was no statistical difference (*p* = 0.147). The number of astrocytes in the hippocampus of the HF + TS group was still higher than the control group (*p* = 0.044) (Fig. 5).

### Teasaponin improved recognition memory and increased BDNF levels in the hippocampus of HF diet-induced obese mice

**Figure 6:**
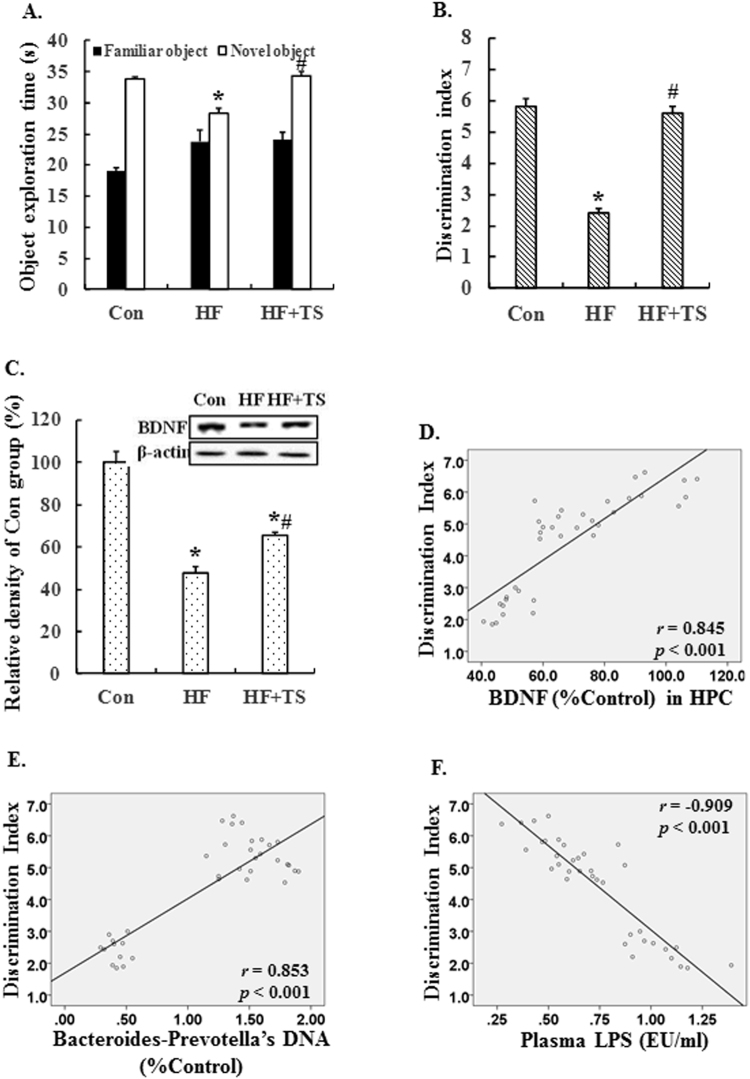
The object exploration time (**A**) and the discrimination index (**B**) in the novel object recognition test of the control group (Con), high-fat diet group (HF) and HF with teasaponin treatment group (HF + TS). (**C**) The level of BDNF expression in the hippocampus of mice (n = 12 per group). The discrimination index in novel object recognition was positively correlated with BDNF in the hippocampus (**D**) and *Bacteroides-Prevotella* spp. DNA in cecal contents (**E**), and was negatively correlated with plasma LPS (**F**). *p < 0.05 compared to the Con group, ^#^ *p* < 0.05 compared to the HF group, values are means ± SEM.

To assess whether teasaponin treatment can prevent HF diet-induced recognition memory deficits, we performed a novel object recognition test in mice. During the training session of the test, there was no significant difference in the percentage of time spent exploring the objects in the open-field among the control group, the HF group and the HF + TS group (*p* > 0.05). During the retention session, HF mice showed reduced novel object exploration compared to controls, while the teasaponin treatment restored this to control levels (Control: 33.84 ± 2.58 seconds, HF: 28.25 ± 1.55 seconds, HF + TS: 34.36 ± 2.57 seconds, *p* = 0.001) (Fig. 6A). The discrimination index was further analyzed to assess recognition memory. The HF diet decreased the discrimination index by 58.62% compared to the control group (*p* < 0.001), while the teasaponin treatment increased the discrimination index by 133.33% compared with the HF group (*p* < 0.001) without significant difference compared with control group (Fig. 6B). These results show that teasaponin treatment improved recognition memory deficits caused by a HF diet.

We then measured hippocampal BDNF levels, which are important for synaptic plasticity and learning and memory. Compared with the control group, the BDNF level of the HF group was significantly lower (*p* < 0.001) (Fig. 6C). Importantly, the teasaponin treatment significantly increased the BDNF level in the hippocampus of the HF + TS group compared to the HF group (*p* = 0.011), but the BDNF level in the HF + TS group was still lower (*p* < 0.001) than the control group (Fig. 6C). There was a significant positive correlation between the hippocampal BDNF level and the discrimination index of the novel object recognition test (*r* = 0.845, *p* < 0.001) (Fig. 6D). Furthermore, the discrimination index was also positively correlated with *Bacteroides-Prevotella* spp. DNA in cecal contents (*r* = 0.853, *p* < 0.001) (Fig. 6E) and was negatively correlated with the plasma LPS level (*r* = −0.909, *p* < 0.001) (Fig. 6F).

### Teasaponin reduced body weight and improved glucose tolerance in HF diet-induced obese mice

**Figure 7:**
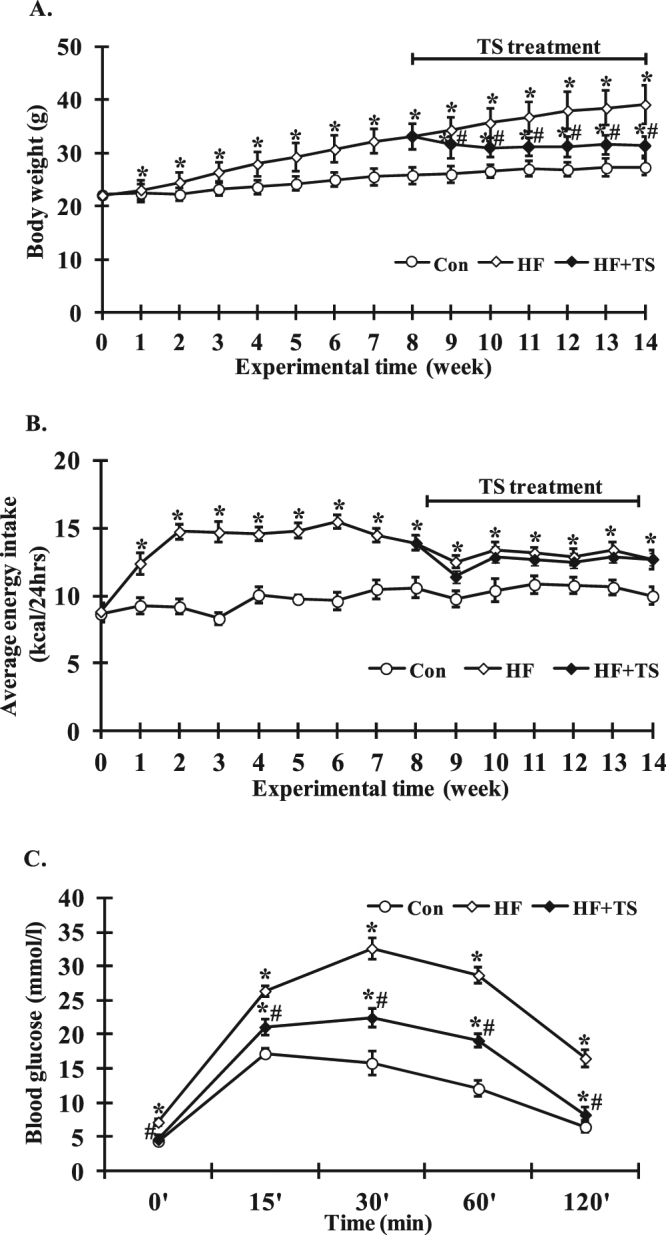
Body weight (**A**), energy intake (**B**) and glucose tolerance (**C**) of the control group (Con), high-fat diet group (HF) and HF with teasaponin treatment group (HF + TS). *p < 0.05 compared to the Con group, ^#^ *p* < 0.05 compared to the HF group, values are means ± SEM.

The body weight in HF groups was significantly higher than that of the control group (HF: 33.11 ± 2.41 g, Control: 25.80 ± 1.56 g, *p* < 0.05) after 8 weeks of HF diet (Fig. 7A). After one week of treatment, the teasaponin group began to show a decrease in body weight. After the teasaponin treatment for 6 weeks, the final body weight was significantly lower in the HF + TS group than the HF group (HF + TS: 31.32 ± 1.87 g, HF: 39.14 ± 3.67 g, *p* = 0.038), however the body weight in the HF + TS group remained higher than the control group (HF + TS: 31.32 ± 1.87 g, Control: 27.33 ± 1.54 g, *p* < 0.001) (Fig. 7A). There was no difference in energy intake during the teasaponin treatment period between HF + TS and HF group (all *p* > 0.05) (Fig. 7B). Furthermore, teasaponin significantly improved glucose tolerance in the HF diet-induced obese mice (Fig. 7C). The fasting glucose level of the HF + TS group and the control group was significantly lower than the HF group (all *p* < 0.05), while there was no difference between the HF + TS group and the control group (*p* = 0.326). The blood glucose level of the teasaponin treatment group significantly decreased at 15, 30, 60, and 120 minutes compared with the HF group (all *p* < 0.05), but they were still higher than those of the control group (all *p* < 0.05).

## Discussion

Our results show that a long-term teasaponin treatment improved gut microbiota, suppressed endotoxin in plasma, enhanced object recognition memory, inhibited hippocampal inflammation and gliosis, and increased BDNF in the hippocampus in HF diet fed mice. Previously, tea or derivative powders have been shown to improve fecal microbiota and have anti-inflammatory benefits in both human and animal studies^21,22^. Moreover, clinical and animal studies have shown that tea improved cognitive function and prevented impairment of learning and memory^23–25^. In the present study, treatment with teasaponin, an important active ingredient of tea, improved microbiota and enhanced memory for object recognition in HF diet fed mice. Therefore, the effect of teasaponin on manipulation of the gut microbiota may contribute to the ability of tea to improve cognitive function and memory.

Compelling evidence suggests there is an association between gut microbiota, HF diet and body weight regulation^4,12^. The ratio of the two dominant gut phyla, Firmicutes and Bacteroidetes, increases with obesity in human studies and animal experiments^4,14,29^. A HF diet for 8 weeks post-weaning has been shown to change the microbiota, decrease the overall bacterial abundance, and increase the ratio of Firmicutes to Bacteriodetes in mice^4^. Furthermore, alteration of gut microbiota induced by increased nutrient load has been directly correlated with stool energy loss in lean individuals, as evidenced by an increase in Firmicutes and a decrease in Bacteroidetes which were associated with an increased energy harvest in subjects with a greater fractional decrease in stool energy loss^12^. In our study, we found that teasaponin prevented a HF diet induced alteration of microbiota at the species level, especially increasing *Bacteroides-Prevotella* spp. and decreasing *Lactobacillus* spp belonging to Bacteroidetes and Firmicutes respectively^30^. Overall these findings suggest that the prebiotic-like effects of teasaponin on microbiota may contribute to decreased energy accumulation and body weight gain with stool energy loss following improving microbiota.

In the present study, teasaponin treatment alleviated the hyper-endotoxemia in HF diet-induce obese mice. Previously, the plasma LPS level was closely correlated with changes in the Gram-negative Bacteroides-like intestinal bacteria that reside within the Bacteroidetes phylum^31^. We found elevated plasma LPS and decreased Gram-negative bacterium *Bacteroides-Prevotella* spp. and *Desulfovibrios* spp. DNA levels in HF diet-induced obese mice. An elevated plasma LPS level has been related to the over-production of LPS in the gut by the death of Gram-negative bacteria and their translocation into the bloodstream via an increased intestinal permeability in HF diet-induced obesity^32^. Therefore, a reduction in Gram-negative bacterium, as observed in the present study, may over-release LPS leading to hyperendotoxemia in the HF diet-induced obese mice. Importantly, dietary teasaponin decreased plasma LPS in these obese mice, perhaps by preventing the decrease of Gram-negative bacterium (including *Bacteroides-Prevotella* spp. and *Desulfovibrios* spp.) induced by a HF diet at the same time. These data strongly suggest that teasaponin affected the intestinal microbiota and was responsible for the attenuation of metabolic endotoxemia in HF diet-induced obesity. A HF diet has previously been shown to elevate LPS levels in colonic fluid to activate pro-inflammatory cytokines (TNF-α, IL-6 and IL-1β) in the colon^33^. Our study showed that a chronic high-fat diet induced pro-inflammatory M1 macrophage accumulation in the colon of obese mice accompanied by increased levels of TNF-α, IL-6 and IL-1β, M1 macrophage produced pro-inflammatory cytokines^34,35^. Importantly, this present study shows that dietary teasaponin significantly ameliorated M1 macrophage accumulation in the colon of HF diet-induced obese mice.

We report that *Lactobacillus* spp. and *Bifidobacterium* spp. were increased and *Desulfovibrios* spp. was decreased following a HF diet. While *Desulfovibrios* spp. has been found to be increased in a previous study of HF diet-induced obesity^36^, *Lactobacillus* spp. and *Bifidobacterium* spp have been reported to decrease following HF diet in rodents^13,36–38^. This contrast may be due to differences in food composition (percentage of fat, type of fatty acids or feeding period) or experimental methods (feeding HF diet or inoculating fresh feces from HF diet fed mice) between the studies. For example in P. D. Cani’s study, the HF diet contains 72% of the energy from fat which is higher than that used in our study (60%)^37^. In Kim’s study, inoculation with HF diet faeces resulted in a reduced number of Bifidobacteria colonies in mice^13^. Our finding of increased *Lactobacillus* spp in the gut microbiome of mice after HF diet, is consistent with a human study which reported a significantly higher concentration of Lactobacillus species in obese patients than in lean controls or anorexic patients^39^. Further, it was reported that specific Lactobacillus in human feces, such as Lactobacillus reuteri or Lactobacillus sakei, were positively correlated with body mass index in 263 individuals, including 134 obese, 38 overweight, 76 lean and 15 anorexic subjects^40^, proving support for an association between increased Lactobacilius and increased body weight. It is reported that *Bifidobacterium* spp is significantly increased in HF diet for 4 weeks in mice and ob/ob mice^41^, which is in agreement with our observations. Furthermore, many different species and strains of *Lactobacillus* spp and *Bifidobacterium* spp contribute to weight gain in infants. For example, Bifidobacterium lactis was significantly associated with increased weight gain in infants born to mothers with HIV infection^42^. Lactobacillus acidophilus, Lactobacillus fermentum and Lactobacillus ingluviei administration significantly increases weight gain in infants^43^. Therefore, the role of different species or strain of *Lactobacillus* spp. and *Bifidobacterium* spp. in weight gain require further clarification.

Consistent with a previous study that teasaponin affects the rumen fermentation and increases microbial mass yield *in vitro* ^44^, the present study showed that teasaponin affects gut microbiota in HF diet-induced obese mice. The exact mechanism underlying this is not clear, however, teasaponin improves the enzymatic hydrolysis of dietary fibre (such as glucan and lignin), which are microbiota-accessible carbohydrates^45^. Teasaponin can also be degraded by cellulolytic enzymes and release glucose, which could be utilized by microbiota to promote saccchrification and fermentation^45^.

Previously, systemic LPS administration activated microglia and increased expression of pro-inflammatory factors in the hippocampus of mice^16^. We found that HF diet induced hippocampal inflammation in obese mice with hyper-endotoxemia. Therefore, the systemic accumulation of toxins, induced by a HF diet may contribute to inflammation in the hippocampus. It has been reported that activated microglial cells release pro-inflammatory cytokines IL-1 and TNF-α, which contribute to the neurodegenerative process^46^. The activation of astrocytes is known to induce innate and adaptive immunity^47^ and astroglial responses were assessed in the present study by GFAP immunostaining. Our findings showed that HF diet promoted the expression of GFAP and Iba-1 in hippocampus suggesting an excessive infiltration of glial cells and possible gliosis. Importantly, dietary teasaponin constrained the activation of Iba-1, but not GFAP, induced by the HF diet. It is known that astrocytes contain glycogen and are capable of glycogenesis. Therefore, teasaponin only inhibited the inflammatory response in microglia, but did not affect astrocytes in the hippocampus. Hippocampal inflammation is highly associated with recognition memory impairment^48^. In the present study, teasaponin significantly improved recognition memory in a novel object testing procedure with inhibition of microgliosis and inflammation in the hippocampus. Therefore, teasaponin, in preventing hyperendotoxemia and hippocampal inflammation, may contribute to recognition memory improvement.

TLR4 is the receptor for LPS and expressed in microglia, astrocytes and neurons^49,59^. Activation of TLR4 signaling inhibits neurogenesis^50^, while deficiency of TLR4 dramatically ameliorates neuroinflammation in the hippocampus of C57BL/6 mice^51^. As a critical adapter protein for TLR4, MyD88 leads to an activation of downstream nuclear factor-κB (NFκB)/JNK and subsequent production of pro-inflammatory cytokines implicated in neurotoxicity^52–54^. In this study, dietary teasaponin prevented the activation of the TLR4-My88-JNK/NFκB inflammatory signaling pathway and an over-expression of pro-inflammatory cytokines in the hippocampus induced by a chronic HF diet. Previous studies have demonstrated that inflammation decreases the expression of BDNF in the rat hippocampus^55,56^ and reduced hippocampal BDNF is associated with recognition memory impairment; increasing hippocampal BDNF has been shown to improve recognition memory^57^. In the present work, teasaponin increased BDNF levels in the hippocampus of obese mice, which may contribute to the improvement in recognition memory in these mice. Furthermore, in the present study, there was a significant positive correlation between the level of *Bacteroides-Prevotella* spp. and cognitive ability as measured by the discrimination index in the novel object recognition test. Indeed, a recent study reports that infants with high levels of gut *Bacteroides* at 1 year of age show higher cognitive ability at 2 years old^58^. Overall, dietary teasaponin may improve microbiota-gut-brain function and cognition.

In conclusion, this study demonstrated that dietary teasaponin reinstalls gut microbiota, and reduces gut mucosal inflammation and hyperendotoxinemia in chronic HF diet-induced obese mice. These peripheral beneficial effects are accompanied by a reduction of CNS hippocampal microglia activation, an increase of BDNF production, and an improvement of cognitive function in obese mice. The behavioral and neurochemical improvements suggest that teasaponin could be a promising strategy to improve gut-brain axis for the prevention of HF diet-induced obesity and associated cognitive decline. This study demonstrated the beneficial effect of teasaponin on gut microbiota using qRT-PCR. To further characterize detailed changes of gut microbiota composition, future studies should use 16S rRNA sequencing or metagenomic sequencing. Furthermore, to elucidate the effects on the brain-gut axis, more candidate species of microbiota, which may be involved in processes such as amyloid secreting, γ-aminobutyric acid biosynthesis, short-chain fatty acids biosynthesis, histone deacetylation (HDAC) and G-protein-coupled receptors, should be analysed.

## Methods

### Animals and treatments

Thirty-six C57BL/6 J male mice (8 weeks old) were purchased from the Australian Bio-Resource Centre (Moss Vale, NSW), and housed in environmentally controlled conditions (temperature 22 °C, 12 hour light/dark cycle). Twelve mice received a lab chow diet as a control (Con group). Twenty-four mice received a HF diet (HF group) containing 60% of energy from fat (SF13-092; Specialty Feeds, Glen Forrest, WA). After 8 weeks, the twenty-four mice fed a HF diet were divided into two groups: twelve mice continued to be fed the HF diet, and the other twelve mice were fed this diet with the addition of 0.5% teasaponin^60^ (HF + TS group) for 6 weeks. Teasaponin (96%, C_57_H_90_O_26_, MW = 1200) was purchased from the Aladdin Chemistry Co. Ltd, China. Body weight was measured on the last day in every week. Food intake was recorded on the first day in every week. A weighed amount of fresh diet was given at the beginning of the dark cycle. The remaining food in the cage plus spillage were collected and weighed 24 hours later. After 6 weeks of treatment, the novel object recognition test and intraperitoneal glucose tolerance test (IPGTT described below) were carried out. All mice were asphyxiated in chambers prefilled with CO_2_ four days after the tests were carried out. Plasma, cecal contents, colon tissue and hippocampus tissues were collected, snap frozen and stored at −80 °C for further analyses as detailed below. The study was approved by the University of Wollongong Animal Ethics Committee (AE13/11) and all animal experiments were conducted in compliance with the National Health and Medical Research Council Australian, Code of Practice for the Care and Use of Animals for Scientific Purposes (2004).

### Real-time PCR (qRT-PCR) to quantify microbial species from cecal content

DNA from cecal contents were extracted by the QIAamp DNA Stool Minikit (QIAGEN, Germany) according to the manufacturer’s instructions. Group-specific primers based on 16S rDNA sequences PCR assay were forward *Bacteroides-Prevotella*, GAGAGGAAGGTCCCCCAC; reverse *Bacteroides-Prevotella*, CGCTACTTGGCTGGTTCAG; forward *Desulfovibrios*, CCGTAGATATCTGGAGGAACATCAG; reverse *Desulfovibrios*, ACATCTAGCATCCATCGTTTACAGC; forward *Lactobacillus*, GAGGCAGCAGTAGGGAATCTTC; reverse *Lactobacillus*, GGCCAGTTACTACCTCTATCCTTCTTC; forward *Bifidobacterium*, CGCGTCTGGTGTGAAAG; reverse *Bifidobacterium*, CCCCACATCCAGCATCCA. Quantitative real-time PCR was performed in a 20-μL final reaction volume using a SYBR green I master in a Lightcycler 480 (F. Hoffmann-La Roche Ltd, Switzerland). Amplification was carried out with 40 cycles of 95 °C for 5 seconds, 60 °C for 10 seconds, and 72 °C for 10 seconds. Each assay was performed in duplicate in the same run. The level of expression for each gene was calculated using the comparative threshold cycle value (Ct) method, using the formula 2^−ΔΔCt^ (where ΔΔCt = ΔCt sample–ΔCt reference). The final results were expressed as normalized fold values relative to the normal group as described previously^61^.

### Lipopolysaccharide (LPS) determination

The concentration of plasma LPS was measured by enzyme-linked immunosorbent assay (LAL assay kit, Hycult Biotech, The Netherlands). The absorbance at 405 nm was measured with a spectrophotometer. The measurable concentration ranges was from 0.04 to 10 EU/ml. All samples for LPS measurements were performed in duplicate.

### Western blotting

Hippocampi were dissected and homogenized in NP-40 lysis buffer. The following antibodies were used: MyD88 (1:1000 dilution; sc-74532), NFκB (1:1000 dilution; sc-7178), p-JNK (1:1000 dilution; sc-81502), IL-1β (1:1000 dilution; sc-7884), BDNF (1:1000 dilution; sc-20981) and IL-6 (1:1000 dilution; sc-7920) all purchased from Santa Cruz Biotechnology (Santa Cruz, CA); TNF-α (1:1000 dilution; #11948) and TLR4 (1:1000 dilution; #2219) were purchased from Cell Signaling Technology (Beverly, MA). The bands corresponding to the proteins of interest were scanned and band densities were analyzed using the automatic imaging analysis system, Quantity One (Bio-Rad Laboratories, Hercules, CA). All quantitative analyses were compared with the control group.

### Immunohistochemistry

As described previously^62^, fixed colon tissues were sectioned at 5 µm and incubated overnight at 4 °C with primary antibodies. Sections were then incubated consecutively with secondary antibodies for 2 hours at room temperature. The sections were then washed and incubated with streptavidin-HRP polymer conjugate (Sigma-Aldrich, Sydney, Australia). The sections were then washed and developed using the ImmPACT DAB peroxidase substrate kit (Vector laboratories Inc., Burlingame, USA) and counterstained with haematoxylin (POCD Scientific, Artarmon, Australia). Six fields from three sections of each mouse were captured. The immunohistochemical staining F4/80, CD11c, and CD206 were quantified as a percentage of positive area per image by Image J software. The immunoreactivity is quantified as the percentage of pixels in an area of interest that have intensity greater than the background.

Fixed hippocampus tissues were sectioned at 35 µm. The sections were washed 3 times with Tris-Buffered Saline (TBS) for 10 minutes, and then washed in 1% H_2_O_2_ in phosphate buffer saline (PBS) for 30 minutes. After three washings with PBS for 5 minutes, the sections were blocked with 5% normal horse serum for 1 hour and incubated overnight at 4 °C with primary antibodies. Then sections were washed 3 times with PBS and Tween 20 (PBST) and incubated consecutively with secondary antibodies for 2 hours at room temperature. The sections were then washed and incubated with streptavidin-HRP polymer conjugate (Sigma-Aldrich Pty. Ltd, Sydney, Australia) for 1 hour at room temperature. The sections were then washed and developed using the ImmPACT DAB peroxidase substrate kit. Six fields from three sections of each mouse were viewed under a Leica microscope and digital photographs were captured. Iba1 and GFAP were used to evaluate the activated phenotype of microglia and astrocytes according to Iba1 and GFAP positive staining and cell morphology. Positive cells were manually counted using Photoshop (Adobe).

### Novel object recognition test

Recognition memory was assessed by performing a novel object recognition test based on our group’s previous studies^5^. Briefly, mice received a 5 minutes habituation in an empty open field arena (a white open-field square box, 55 cm × 55 cm × 35 cm). During the training session, two identical objects (A) were placed at opposing corners of the box, 5 cm from the adjacent wall. Each mouse was then placed in the middle of the open-field box and left to explore the objects for 5 minutes, and the percentage of time spent exploring the two identical objects in the open-field box was calculated (time spent on the two identical objects divided by 5 minutes). For the retention session, one familiar object (A) was replaced with one novel object (B) and measurements were taken according to how much time each mouse spent at each object as per the training session. Following a 90-minute inter-trial interval, we returned mice to explore for another 5 minutes. Novel object exploration time and the discrimination index (DI = [(Novel Object Exploration Time/Total Exploration Time) − (Familiar Object Exploration Time/Total Exploration Time)] × 100) were used to evaluate the recognition memory of the mice^63^.

### Intraperitoneal glucose tolerance test

All mice were fasted overnight before a glucose tolerance test was performed to assess glucose clearance, following an intraperitoneal injection of glucose (0.5 g/kg; Sigma-Aldrich, St Louis, MO, USA). Blood samples were taken from the tail vein at 0, 15, 30, 60 and 120 minutes following the injection of glucose. Blood glucose was measured using an Accu-Chek glucometer (Roche Diagnostics GmbH Mannheim, Germany).

### Statistical analysis

The statistical package SPSS 20 (SPSS, Chicago, IL) was used to analyze data. Data was first tested for normality before differences among the Con, HF and HF + TS groups were determined using one-way analysis of variance (ANOVA). This was followed by the post hoc Tukey-Kramer honestly significant difference (HSD) test for multiple comparisons among the groups. A *p* value of <0.05 was considered to be statistically significant. Values were expressed as mean ± SEM. Pearson’s correlations were used to examine the relationship between the discrimination index in the novel object recognition test and plasma LPS and BDNF levels in the hippocampus.
